# Effects of a Social Media Intervention on Vaping Intentions: Randomized Dose-Response Experiment

**DOI:** 10.2196/50741

**Published:** 2024-03-12

**Authors:** William Douglas Evans, Jeffrey Bingenheimer, Jennifer Cantrell, Jennifer Kreslake, Shreya Tulsiani, Megumi Ichimiya, Alexander P D'Esterre, Raquel Gerard, Madeline Martin, Elizabeth C Hair

**Affiliations:** 1 Milken Institute School of Public Health The George Washington University Washington, DC United States; 2 New York University School of Global Public Health New York, NY United States; 3 Truth Initiative Washington, DC United States

**Keywords:** randomized controlled trial, e-cigarettes, vaping, nicotine, tobacco control, social media, dose-response effects

## Abstract

**Background:**

e-Cigarette use, especially by young adults, is at unacceptably high levels and represents a public health risk factor. Digital media are increasingly being used to deliver antivaping campaigns, but little is known about their effectiveness or the dose-response effects of content delivery.

**Objective:**

The objectives of this study were to evaluate (1) the effectiveness of a 60-day antivaping social media intervention in changing vaping use intentions and beliefs related to the stimulus content and (2) the dose-response effects of varying levels of exposure to the intervention on vaping outcomes, including anti-industry beliefs, vaping intentions, and other attitudes and beliefs related to vaping.

**Methods:**

Participants were adults aged 18 to 24 years in the United States. They were recruited into the study through Facebook (Meta Platforms) and Instagram (Meta Platforms), completed a baseline survey, and then randomized to 1 of the 5 conditions: 0 (control), 4, 8, 16, and 32 exposures over a 15-day period between each survey wave. Follow-up data were collected 30 and 60 days after randomization. We conducted stratified analyses of the full sample and in subsamples defined by the baseline vaping status (never, former, and current). Stimulus was delivered through Facebook and Instagram in four 15-second social media videos focused on anti-industry beliefs about vaping. The main outcome measures reported in this study were self-reported exposure to social media intervention content, attitudes and beliefs about vaping, and vaping intentions. We estimated a series of multivariate linear regressions in Stata 17 (StataCorp). To capture the dose-response effect, we assigned each study arm a numerical value corresponding to the number of advertisements (exposures) delivered to participants in each arm and used this number as our focal independent variable. In each model, the predictor was the treatment arm to which each participant was assigned.

**Results:**

The baseline sample consisted of 1491 participants, and the final analysis sample consisted of 57.28% (854/1491) of the participants retained at the 60-day follow-up. We compared the retained participants with those lost to follow-up and found no statistically significant differences across demographic variables. We found a significant effect of the social media treatment on vaping intentions (β=−0.138, 95% CI −0.266 to −0.010; *P*=.04) and anti-industry beliefs (β=−0.122, 95% CI 0.008-0.237; *P*=.04) targeted by the intervention content among current vapers but not among the full sample or other strata. We found no significant effects of self-reported exposure to the stimulus.

**Conclusions:**

Social media interventions are a promising approach to preventing vaping among young adults. More research is needed on how to optimize the dosage of such interventions and the extent to which long-term exposure may affect vaping use over time.

**Trial Registration:**

ClinicalTrials.gov NCT04867668; https://clinicaltrials.gov/study/NCT04867668

## Introduction

### Background

e-Cigarettes were the most commonly used tobacco product among young adults in the United States between 2014 and 2019 [1]. In 2019, the current use of cigarettes and e-cigarettes was 4.5% and 14% [2], respectively. Although e-cigarette use among this population has decreased in recent years, use prevalence still remains at concerning levels. Moreover, the most popular e-cigarette brands contain high levels of nicotine, an addictive substance that may harm the developing brain of young adults [3-6]. The use of e-cigarettes has also been associated with worsened lung health and mental well-being [7-11].

Digital media, including social media platforms, have become a part of our daily lives, particularly among young adults. The use of any social media site by adults aged 18 to 29 years has been consistently >80% since 2011, and many people spend several hours a day on these sites (Pew Research Center, 2021) [12]. Because of its ubiquity and potential for influence, digital media can be a valuable or harmful tool for population-level behavior change. Thus, there is a great need for more research on the relationship between digital media and health behaviors, social norms, and social networks. Although research is being conducted to determine what digital media as an intervention tool would look like, how it works, and how effective it is [13,14], these studies have only scratched the surface [15].

The importance of digital media interventions is growing in many health behavior subject areas, including nicotine and tobacco use research. Mass media campaigns have been proven to be effective in creating positive changes in smoking-related attitudes, intentions, and behaviors [16]. More recent research also supports the use of media campaigns to address the aforementioned rapid increase in and continued use of e-cigarettes among youth and young adults [17]. Digital strategies will be central to future campaigns. A recent systematic review of digital behavior change interventions by Ichimiya et al [18] identified 298 relevant articles; 19 of those were for nicotine and tobacco interventions.

### Prior Work

Digital media intervention research is currently a small, growing, and highly important field, given the shift in nicotine behavior change campaigns from traditional mass media such as television to digital platforms [19]. These strategies have the potential to change social norms (ie, beliefs among a population about what is widespread behavior and what is socially sanctioned or required) [20] about behaviors such as vaping. Social norms may be influenced by, for example, e-cigarette companies’ social media platforms [21], which normalize and effectively promote use among a peer group such as young adults [19]. At the same time, antivaping social media may create a new social norm that vaping is uncommon and less socially accepted among the peer group. Theoretically, the effect of such social media campaigns may be to promote a social norm such as the avoidance of nicotine and tobacco products [20].

Specifically, given the relatively small number of studies found in the systematic review of digital tobacco behavior change interventions by Ichimiya et al [18], there is a need to rigorously test the effects of antivaping social media content on outcomes. Many large-scale campaigns, such as those run by the Food and Drug Administration (eg, Real Cost) and the Truth Initiative (the Truth campaign), are currently using digital content as part of their overall behavior change strategies, but little is known about their mechanisms of change, and the published research does not include randomized trials [18]. Research is needed to build and test theories of change for such campaigns using randomized experimental methods.

Furthermore, this study builds on recent studies using a social media–based data collection platform for random assignment studies [14]. The use of social media recruitment, chatbots for survey delivery, and retargeting technology for intervention delivery and follow-up (FU) have been proven feasible and produce short-term effects on content (eg, advertising) exposure. This study aims to test these methods in a randomized controlled dose-response experiment.

### Study Aims

In this study, our goal was to determine whether a social media intervention delivered through an experimental design would have a positive effect on young adult vaping outcomes. We aimed to disseminate the intervention on participants’ Facebook and Instagram news feeds in the form of an antivaping campaign consisting of 4 videos, each 15 seconds in length, drawn from previous Truth Initiative content and aimed at young adults aged 18 to 24 years. Participants answered 1 preintervention survey and 2 postintervention surveys on the same platform, Facebook Messenger (FM; Meta Platforms). A chatbot was used to execute the surveys and keep participants engaged over the course of the 60-day study period.

### Hypothesis

We tested the hypotheses that exposure to antivaping social media content measured through a social media–based survey would reduce vaping use intentions at the 60-day FU (FU2; hypothesis 1) and increase antivaping industry beliefs at the FU2 (hypothesis 2). We also examined 1 research question: would treatment assignment be associated with lower use intentions and higher antivaping beliefs (ie, a dose-response effect)?

## Methods

### Study Design

The study design was a randomized controlled experiment with 4 treatment arms and a no-exposure control arm. Using the Virtual Lab platform, participants were recruited into the study (details under *Data Collection and Measures* section), delivered a baseline survey, and then randomized to 1 of the 5 conditions. The design was to achieve a specific number of impressions per arm as follows: 0 (control), 4, 8, 16, and 32 over a 15-day period between each survey wave. Impressions are defined as the number of views of a social media post by a study participant [22].

There were 3 survey waves: baseline, 30-day FU (FU1), and FU2. The aim was to collect sufficient participants within each wave to have sufficient power to detect a treatment effect of the intervention video content exposure on vaping intentions at FU2. The final baseline sample consisted of 1491 participants divided into 5 study conditions: FU1 consisted of 70.28% (1048/1491) of the participants, and FU2 consisted of 57.28% (854/1491) of the participants.

### Intervention Content

The intervention content consisted of four 15-second videos drawn from a previous web-based Truth Initiative campaign called *Tested on Humans*. The main themes of the videos were that vaping companies do not know the health and other impacts of using e-cigarettes and that they are “testing” their products on human beings. This is consistent with an “anti-industry” countermarking approach to nicotine and tobacco campaigns, which has been used successfully in the past [23,24]. The campaign was not publicly active during this study. We chose this content because it was designed for social media distribution, focused on preventing vaping, and was not currently active.

Following baseline, videos were promoted in the live Facebook and Instagram feeds of treatment arm participants in a randomized order and combinations to achieve the targeted impressions for each arm (ie, an average number of impressions per condition). For example, the “low” exposure arm was designed to obtain 4 impressions that would receive a randomly ordered assignment of each video 1 time, the next highest exposure arm (8 impressions) was designed to obtain the videos in random order 2 times, and so on. The actual number of impressions per group varied because of the time of the intervention delivery and was measured at the group level because of the confidentiality restrictions Facebook and Instagram place on publicly available user data (ie, the exact number of impressions by an individual user is not available, only by the study condition). This resulted in the use of a 5-level variable corresponding to the 5 treatment arms of increasing intended impressions (arm 1=0 impressions, arm 2=4 impressions, arm 3=8 impressions, arm 4=16 impressions, and arm 5=32 impressions).

The study was implemented by Virtual Lab, a social media–based data collection and intervention content delivery platform [25]. Participants were recruited via Facebook and Instagram advertisements. When a potential participant clicked on a study advertisement, they were asked a series of screening questions using a FM chatbot. Eligible participants were US residents aged 18 to 24 years within the stratified subgroups, with 49.97% (911/1823) of them being current vapers. The participants were asked to provide informed consent and participate in the study through an FM survey delivered by the chatbot. After completing the baseline questionnaire, the participants were randomized to the study condition, received any relevant content over time, and were invited to complete the FU questionnaires.

**Figure 1:**
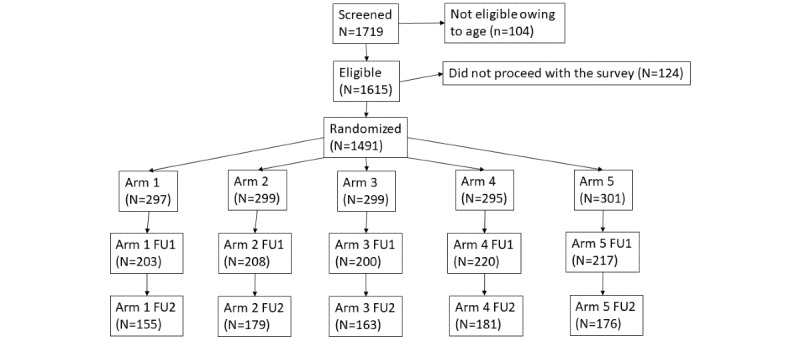
CONSORT (Consolidated Standards of Reporting Trials) diagram. FU1: 30-day follow-up; FU2: 60-day follow-up.

We used the CONSORT (Consolidated Standards of Reporting Trials) checklist when writing our report [26]. The study design and recruitment procedures are summarized in Figure 1. Note that the total retained sample at FU was 57.28% (854/1491), but the sample sizes for some analyses varied because of participant response patterns.

### Ethical Considerations

This study was reviewed and approved as not greater than minimal risk human participants research by the George Washington University Institutional Review Board (IRB) on August 5, 2020(IRB number NCR202837). Through the FM chatbot, participants read an IRB-approved statement informing them about the purposes and nature of the research. By clicking on a button to proceed with the survey, they provided consent to participate. All data used in this study have been deidentified and stored following the IRB-approved procedure to ensure confidentiality. The participants received a US $10 e-gift card as compensation for each survey completed.

### Data Collection and Measures

Similar to a pilot study reported by Tulsiani et al [14], we worked with Virtual Lab to implement the study and collect data [25]. The study team created a Facebook business account called “Digital Health Research” to recruit participants and manage data collection and a second account, “Consumer Consciousness,” to run the target advertisements on the enrolled participants’ Facebook and Instagram news feeds. The recruitment advertisements were served to people aged 18 to 24 years who were located in the United States. The advertisements used the text, “Take a 15 minute survey, get paid $10.” After the participants clicked on the study’s advertisement, they were sent a message via FM inviting them to participate in the study.

The survey was delivered as a series of individual chats through FM using a chatbot. The survey consisted of 40 items drawn from the tobacco control and campaign evaluation literature [27]. For this study, we used a subset of the items contained in the survey, following our study objectives. All items were measured on a 5-point agreement scale, except where noted (strongly agree to strongly disagree).

Our primary end point was future vape intentions, operationalized as the average of responses to 2 items, which were each answered on a 5-point agreement scale: “Thinking about the future, if one of your best friends offered you an e-cigarette/vape (even one or two puffs) in the coming year, would you smoke it?” and “Do you think you will use an e-cigarette/vape (even one or two puffs) in the next year?” Our secondary end point was anti-industry sentiment, measured as the average of responses to 2 items, also on a 5-point agreement scale: “Vape companies make me angry” and “I am willing to stand up with others against vape companies.” Our measures of vaping intentions and anti-industry sentiment are both taken from the second FU survey. Finally, we examined self-reported advertisement exposure. For each of the 4 advertisements, the participants were asked, “Overall, about how many times to do you think you’ve seen this ad? 1-2 times; 3-5 times; more than 5 times.” Responses were recoded to approximate the average value for each category (“Never”=0, “1-2 times”=1.5, “3-5 times”=4, and “>5 times”=6), and an average value across each of these 4 advertisements was calculated to generate an average value of reported advertisement exposure. As we were interested in cumulative exposure, the value for both time points was averaged.

### Data Analysis

To investigate our hypotheses, a series of multivariate linear regressions were conducted in Stata 17 (StataCorp). To capture the dose-response effect, rather than treating the 5 study arms as 5 independent and nominal groups, we assigned each arm a numerical value corresponding to the number of advertisements delivered to participants in each arm and used this number as our focal independent variable. In each model, the predictor was the treatment arm to which each participant was assigned. The outcome variables for these regressions were self-reported advertisement exposure, anti-industry attitudes and beliefs, and vape use intentions. For each of the multivariate linear regressions, the following covariates were included: race and ethnicity (dummy coded for “non-Hispanic White,” “non-Hispanic Black,” “Hispanic,” and “non-Hispanic other”—“non-Hispanic White” was used as the reference category), gender (dummy coded for “female,” “male,” and “another identity/nonbinary/transgender”—“female” was used as the reference category), age in years, and baseline use of e-cigarettes (dummy coded for “Never User,” “Former User,” and “Current User”—“Never User” was used as the reference category).

We included 57.28% (854/1491) of the participants retained at FU2 in our analysis. We compared the retained participants with those lost to FU (LTFU) and found no statistically significant differences across demographic variables. These results are presented in Multimedia Appendix 1.

In addition, different efficacy levels of the treatment were hypothesized for the participants based on their use status at baseline. Therefore, each of the aforementioned model was also applied to subsamples based on e-cigarette use at the final wave of data collection (“Never users,” “Former Users,” and “Current Users”). We note that these subgroup analyses were not among the original hypotheses of the study and were investigated post hoc. All analyses included the full sample, including those LTFU, following the intention-to-treat principles.

### Power Analysis

We conducted a statistical power analysis to determine an appropriate sample size. Because of our planned dose-response analysis, we used correlation analysis as the basis for our calculations. Specifically, we assumed that the correlation between advertisements delivered in each study arm and vaping intentions at FU2 would be small, as low as 0.1, and used Stata 18 (StataCorp) to calculate that a sample of 783 participants would be needed to provide 80% power for rejecting the null hypothesis at the conventional Cronbach α=0.05 level. Second, based on the results of some pilot studies, we assumed that up to 45% (671/1491 based on our actual sample) of the baseline participants would be LTFU before FU2. Thus, we concluded that the baseline sample should include a minimum of 1423 participants, or approximately 19.11% (285/1423) of participants in each of the 5 study arms. We exceeded our recruitment target in the final sample.

## Results

Table 1 provides the descriptive statistics for the baseline sample categorized by the treatment arm.

**Table 1:** Descriptive statistics by treatment arm (n=854).

			Arm 1 (n=155)		Arm 2 (n=179)		Arm 3 (n=163)		Arm 4 (n=181)		Arm 5 (n=176)		Chi-square (*df*)			*P* value	
**Age (y), n (%)**														19.8 (24)			.71
	18	22 (14.2)		20 (11.2)		20 (12.3)		27 (14.9)		20 (11.4)							
	19	21 (13.6)		24 (13.4)		25 (15.3)		26 (14.4)		19 (10.8)							
	20	18 (11.6)		24 (13.4)		22 (13.5)		27 (14.9)		28 (15.9)							
	21	26 (16.8)		34 (19)		17 (10.4)		23 (12.7)		22 (12.5)							
	22	24 (15.5)		26 (14.5)		22 (13.5)		22 (12.2)		32 (18.2)							
	23	16 (10.3)		28 (15.6)		32 (19.6)		27 (14.9)		23 (13.1)							
	24	28 (18.1)		23 (12.9)		25 (15.3)		29 (16)		32 (18.2)							
**Race and ethnicity, n (%)**														11.48 (12)			.49
	Black, non-Hispanic	8 (5.2)		12 (6.7)		10 (6.1)		18 (9.9)		9 (5.1)							
	Hispanic	36 (23.2)		28 (15.6)		27 (16.6)		35 (19.3)		29 (16.5)							
	Other, non-Hispanic	33 (21.3)		52 (29.1)		49 (30.1)		43 (23.8)		50 (28.4)							
	White, non-Hispanic	78 (50.3)		87 (48.6)		77 (47.2)		85 (47)		88 (50)							
**Sex, n (%)**														4.5 (8)			.81
	Female	109 (70.3)		125 (69.8)		117 (71.8)		127 (70.2)		121 (68.8)							
	Male	45 (29)		47 (26.3)		41 (25.2)		48 (26.5)		48 (27.3)							
	Another identity, nonbinary, or transgender	1 (0.7)		7 (3.9)		5 (3.1)		6 (3.3)		7 (4)							
**Perceived financial situation, n (%)**														11.7			.47
	Lives comfortably	51 (32.9)		63 (35.2)		56 (34.4)		55 (30.4)		58 (33)							
	Meets needs with a little left over	42 (27.1)		64 (35.8)		51 (31.3)		69 (38.1)		54 (30.7)							
	Meets basic expenses	54 (34.8)		39 (21.8)		47 (28.8)		45 (24.9)		54 (30.7)							
	Does not meet basic expenses	8 (5.2)		13 (7.3)		9 (5.5)		12 (6.6)		10 (5.7)							
**e-Cigarette use at baseline, n (%)**														7.8 (12)			.80
	Never users	81 (52.3)		108 (60.3)		91 (55.8)		105 (58)		102 (58)							
	Former users	40 (25.8)		35 (19.6)		42 (25.8)		41 (22.7)		33 (18.8)							
	Current users	33 (21.3)		35 (19.6)		30 (18.4)		34 (18.8)		41 (23.3)							
	Missing	1 (0.7)		1 (0.6)		0 (0)		1 (0.6)		0 (0)							
**Sexual orientation, n (%)**														26.8 (20)			.14
	Heterosexual	96 (61.9)		105 (58.7)		106 (65)		124 (68.5)		105 (59.1)							
	Bisexual	25 (16.1)		38 (21.2)		25 (15.3)		18 (9.9)		34 (19.3)							
	Homosexual	11 (7.1)		12 (6.7)		6 (3.7)		10 (5.5)		12 (6.8)							
	Asexual	3 (1.9)		3 (1.7)		8 (4.9)		3 (1.7)		1 (0.6)							
	Another sexual orientation	7 (4.5)		6 (3.4)		1 (0.6)		5 (2.8)		6 (3.4)							
	Missing	13 (8.4)		15 (8.4)		17 (10.4)		21 (11.6)		19 (10.8)							

We used a chi-square test to examine any potential differences in demographics between the arms and found no statistically significant differences. Overall, the sample is relatively evenly distributed, with ages ranging from 18 to 24 years, and just <47.95% (715/1491) of the sample is non-Hispanic White. Approximately 7.98% (119/1491) and 16.97% (253/1491) of the sample were non-Hispanic Black and of Hispanic ethnicity, respectively. Approximately 69.01% (1029/1491) of the sample was female, and approximately 65% (969/1491) reported having more than enough income to support themselves. Just >70.09% (1045/1491) reported being heterosexual, with the next largest group reporting being bisexual at just <17.91% (267/1491). At baseline, approximately 20.99% (313/1491) reported current e-cigarette use (meaning within the past 30 d), and 22% (328/1491) reported former ( >30 d ago) use.

Table 2 provides a summary of the models for treatment effects with covariates on vape use intentions by final use status (full sample, never, former, and current users).

Overall, there is a significant treatment effect among current vapers on lower vaping intentions (β=−0.138; *P*=.04) but not in the full sample or other subgroups. We also see effects on lower vaping intentions among Black participants in the full sample and among baseline former and current vapers in the full sample.

Table 3 provides a summary of the models for treatment effects with covariates on anti-industry attitudes and beliefs, which were the main beliefs targeted by the intervention content (ie, beliefs that the e-cigarette industry harms its customers).

**Table 2:** Treatment effects on vape use intentions (N=836)^a^.

			Full sample					Stratified analyses												
			Analysis (n=836)					Never users (n=478)					Former users (n=197)					Current users (n=157)		
			β (95% CI)		*P* value		β (95% CI)			*P* value		β (95% CI)			*P* value		β (95% CI)			*P* value
Treatment			−0.009 (−0.059 to 0.040)		.71		0.024 (−0.026 to 0.074)			.35		0.059 (−0.051 to 0.169)			.29		−0.138 (−0.266 to −0.010)			.04
**Race and ethnicity**																				
	Black, non-Hispanic	0.321 (0.031 to 0.611)		.03		0.072 (−0.221 to 0.364)			.63		0.236 (−0.603 to 1.07)			.58		0.265 (−0.410 to 0.940)			.44	
	Hispanic	0.009 (−0.180 to 0.198)		.93		0.090 (−0.099 to 0.280)			.35		−0.272 (−0.711 to 0.167)			.22		0.081 (−0.407 to 0.568)			.75	
	Other, non-Hispanic	0.078 (−0.092 to 0.249)		.37		0.042 (−0.124 to 0.209)			.62		0.158 (−0.225 to 0.541)			.42		−0.198 (−0.690 to 0.294)			.43	
	White, non-Hispanic	REF^b^		REF		REF			REF		REF			REF		REF			REF	
**Sex**																				
	Female	REF		REF		REF			REF		REF			REF		REF			REF	
	Male	0.099 (−0.058 to 0.256)		.22		0.072 (−0.085 to 0.228)			.37		0.096 (−0.250 to 0.442)			.59		−0.065 (−0.511 to 0.381)			.78	
	Another identity, nonbinary, or transgender	0.264 (−0.144 to 0.672)		.20		0.216 (−0.230 to 0.663)			.34		0.321 (−0.414 to 1.06)			.39		0.401 (−0.951 to 1.75)			.56	
Age (y)			0.002 (−0.033 to 0.037)		.90		−0.023 (−0.058 to 0.012)			.20		−0.010 (−0.089 to 0.069)			.80		0.073 (−0.020 to 0.165)			.12
**Baseline use status**																				
	Never	REF		REF		N/A^c^			N/A		N/A			N/A		N/A			N/A	
	Former	0.737 (0.564 to 0.910)		<.001		N/A			N/A		N/A			N/A		N/A			N/A	
	Current	1.70 (1.52 to 1.88)		<.001		N/A			N/A		N/A			N/A		N/A			N/A	

^a^For the full-sample analyses, N was 836 because of item nonresponse, and N was 832 for the stratified analyses.

^b^REF: reference.

^c^N/A: not applicable.

**Table 3:** Treatment effects on anti-industry attitudes and beliefs (N=838)^a^.

			Full sample				Stratified analyses												
			Analysis (n=838)				Never users (n=479)					Former users (n=198)					Current users (n=157)		
			β (95% CI)		*P* value	β (95% CI)			*P* value		β (95% CI)			*P* value		β (95% CI)			*P* value
Treatment			0.020 (−0.027 to 0.067)		.41	0.007 (−0.052 to 0.067)			.81		−0.065 (−0.164 to 0.033)			.19		0.122 (0.008 to 0.237)			.04
**Race and ethnicity**																			
	Black, non-Hispanic	−0.409 (−0.681 to −0.137)		.003		−0.203 (−0.553 to 0.147)		.26		−0.988 (−1.673 to −0.303)			.005		−0.186 (−0.796 to 0.423)			.55	
	Hispanic	0.025 (−0.153 to 0.204)		.78		−0.064 (−0.290 to 0.162)		.58		0.120 (−0.273 to 0.512)			.55		0.164 (−0.269 to 0.598)			.46	
	Other, non-Hispanic	−0.245 (−0.406 to −0.084)		.003		−0.246 (−0.444 to −0.047)		.02		−0.385 (−0.725 to −0.045)			.03		0.139 (−0.298 to 0.577)			.53	
	White, non-Hispanic	REF^b^		REF		REF		REF		REF			REF		REF			REF	
**Sex**																			
	Female	REF		REF		REF		REF		REF			REF		REF			REF	
	Male	−0.140 (−0.288 to 0.009)		.07		−0.107 (−0.294 to 0.080)		.26		−0.016 (−0.325 to 0.292)			.92		−0.260 (−0.658 to 0.137)			.20	
	Another identity, nonbinary, or transgender	0.003 (−0.383 to 0.022)		.99		−0.080 (−0.613 to 0.454)		.77		0.345 (−0.309 to 0.999)			.30		−0.591 (−1.81 to 0.630)			.34	
Age (y)			−0.011 (−0.044 to 0.022)		.51	−0.009 (−0.051 to 0.032)			.67		−0.031 (−0.100 to 0.039)			.39		0.027 (−0.056 to 0.110)			.52
**Baseline use status**																			
	Never	REF		REF		REF		REF		REF			REF		REF			REF	
	Former	−0.357 (−0.520 to −0.193)		<.001		N/A^c^		N/A		N/A			N/A		N/A			N/A	
	Current	−0.622 (−0.792 to −0.451)		<.001		N/A		N/A		N/A			N/A		N/A			N/A	

^a^For the full-sample analyses, N was 838 because of item nonresponse, and N was 834 for the stratified analyses.

^b^REF: reference.

^c^N/A: not applicable.

Overall, there is a significant treatment effect among current vapers on anti-industry beliefs (β=0.120; *P*=.046) but not in the full sample or other subgroups. In addition, Black participants were more likely to report an intention to vape than non-Hispanic White participants, and current and former vapers were more likely to report an intention to vape than participants who had never vaped.

Table 4 provides a summary of the models for treatment effects with covariates on self-reported advertisement exposure.

There is no main effect of treatment on the reported advertisement exposure among any of the population groups of interest. In addition, we observe higher self-reported advertisement exposure among Black participants in the full sample, and exposure to intervention content was higher among baseline never vapers and baseline current 30-day vapers. It is possible that the lack of relationship between the treatment group and reported advertisement exposure is because of insufficient elapsed time during the intervention period to achieve the intended number of impressions per group, which resulted in participants in the fourth and fifth groups receiving similar levels of impressions. The average impressions delivered per user per treatment arm were as follows: arm 1=0, arm 2=2.293, arm 3=7.708, arm 4=12.718, and arm 5=14.218.

**Table 4:** Treatment effects on self-reported advertisement exposure (N=730)^a^.

			Full sample				Stratified analyses												
			Analysis (n=730)				Never users (n=407)					Former users (n=180)					Current users (n=139)		
			β (95% CI)		*P* value	β (95% CI)			*P* value		β (95% CI)			*P* value		β (95% CI)			*P* value
Treatment			−0.004 (−0.042 to 0.034)		.83	−0.020 (−0.069 to 0.030)			.44		−0.006 (−0.068 to 0.055)			.85		0.066 (−0.035 to 0.167)			.20
**Race and ethnicity**																			
	Black, non-Hispanic	0.312 (0.087 to 0.536)		.007		0.681 (0.392 to 0.969)		<.001		0.143 (−0.263 to 0.549)			.49		−0.430 (−1.04 to 0.183)			.17	
	Hispanic	−0.071 (−0.216 to 0.075)		.34		−0.160 (−0.350 to 0.030)		.10		0.008 (−0.236 to 0.254)			.94		−0.024 (−0.349 to 0.397)			.90	
	Other, non-Hispanic	−0.187 (−0.317 to −0.057)		.005		−0.260 (−0.426 to −0.094)		.002		−0.155 (−0.364 to 0.055)			.15		−0.126 (−0.501 to 0.249)			.51	
	White, non-Hispanic	REF^b^		REF		REF		REF		REF			REF		REF			REF	
**Sex**																			
	Female	REF		REF		REF		REF		REF			REF		REF			REF	
	Male	0.088 (−0.030 to 0.207)		.14		−0.036 (−0.189 to 0.117)		.64		0.106 (−0.084 to 0.296)			.27		0.378 (0.044 to 0.712)			.03	
	Another identity, nonbinary, or transgender	−0.064 (−0.376 to 0.028)		.69		0.167 (−0.308 to 0.643)		.49		−0.124 (−0.512 to 0.265)			.53		−0.257 (−1.27 to 0.753)			.62	
Age (y)			0.001 (−0.026 to 0.028)		.93	−0.007 (−0.042 to 0.027)			.67		−0.012 (−0.055 to 0.031)			.58		0.055 (−0.017 to 0.128)			.13
**Baseline use status**																			
	Never	REF		REF		N/A^c^		N/A		N/A			N/A		N/A			N/A	
	Former	−0.080 (−0.211 to 0.052)		.24		N/A		N/A		N/A			N/A		N/A			N/A	
	Current	0.124 (−0.014 to 0.262)		.08		N/A		N/A		N/A			N/A		N/A			N/A	

^a^For the full-sample analyses, N was 730 because of item nonresponse, and for the stratified analyses, N was 726.

^b^REF: reference.

^c^N/A: not applicable.

## Discussion

### Overview

e-Cigarette use among young adults is a significant public health threat. Use rates dropped early in the COVID-19 pandemic but have seen a resurgence in the later stages of this public health emergency [28]. Innovative strategies to deliver antivaping messages and reduce use intentions and behavior are needed. Given the high levels of social media use among adolescents and young adults, and especially engagement with provaping content [29], interventions using social media are an important intervention channel for experimentation and population-level campaigns.

### Principal Findings

Overall, this study found significant effects in the direction expected for intentions and anti-industry sentiment. Our hypothesis 1 was partially confirmed: there was a significant treatment effect on both anti-industry beliefs and lower vaping intentions, but these effects were limited to current e-cigarette users and were not observed among never or past users or in the full-sample analyses.

This study partially confirmed hypothesis 2: higher levels of treatment (ie, from arms 1 to 5) were associated with the anti-industry beliefs and vaping intentions outcomes of interest. However, we did not observe a direct or dose-response effect of the intervention on content exposure outcomes (ie, awareness of the specific social media posts used as stimulus in the study). This is typically the most proximal outcome resulting from a campaign, and the absence of these anticipated effects deserves further investigation.

One possible explanation is that the experiment did not completely achieve the intended levels of impressions for each study arm. In particular, the level of impression achieved at the highest exposure arms (4 to 5) was quite similar, whereas the intent was to double the number of impressions in each arm. This may be an artifact of the length of time our intervention was in the field, which was only 60 days. Total social media impressions are typically a function of the length of a campaign, and longer study durations may result in more closely matching the intended exposure levels by study condition [30,31].

The observed effects of treatment on anti-industry beliefs are consistent with the content of the intervention, which focused on messages exposing the misinformation and disinformation that e-cigarette companies use in their marketing and the idea that their products and practices are harmful to consumers. This study provides evidence to support the idea that targeted campaign messages can directly impact attitudes and beliefs focused on the content of those messages. Future studies should examine approaches to optimize these observed effects.

The observed effects on lower vape use intentions suggest that there may be a connection between anti-industry beliefs and future use among current users. If young adults believe that the industry is using misinformation and disinformation and selling a harmful product, they may reconsider their current use [32]. This suggests a potential mediation effect of anti-industry beliefs on intentions and potentially on e-cigarette use. The hypothetical pathways of effects should be formally evaluated in future studies.

### Future Directions

One question raised by this study’s findings for vaping intentions and anti-industry beliefs is why we did not see effects among the former and never vapers. However, it is simply difficult to shift beliefs and intentions in those other groups than among current vapers. Alternatively, messages focused on topics and persuasive content other than anti-industry sentiment may be needed for those groups. In addition, selective attention bias (ie, the personal relevance of vaping-related content) suggests that vapers may be more responsive to the antivaping social media content used in this study [33]. Future research should examine these questions.

To fully examine the dose-response effects of social media interventions, longer time durations may be needed, and larger small sample sizes per study arm may be needed, especially given the attrition at FU. Cell sizes between the study conditions were reduced at the second FU, which may have reduced the statistical power to below the levels needed to detect some dose-response outcomes of interest. Previous studies have shown the dose-response effects of anti-industry messaging on vaping-related content exposure and attitudinal outcomes [14].

This study also contributes to the growing literature on public health, social media interventions, and theories of change [34,35]. This study further demonstrates the potential of a social media–based research and intervention delivery platform to build evidence for tobacco control. Future studies should expand on this research with longer-term longitudinal studies capable of potentially detecting treatment effects on vaping use behavior; examine diverse subgroups of interest, including high-risk groups for e-cigarette use; and examine multiple types of social media content. Finally, the demonstrated effects of social media on intentions and other outcomes related to vaping should be considered when formulating a tobacco control policy, including recommendations for effective comprehensive prevention and cessation interventions [36].

### Limitations

Finally, this study has some limitations. First, it was conducted over a relatively short period (60 d), and thus, only intermediate outcomes were evaluated. In addition, the time duration may have limited our ability to fully generate the intended differences in the objective impressions created within each study arm. We observed a substantial LTFU (>40%) at the second FU survey. Future social media studies should make extensive efforts to limit LTFU, especially when following participants over longer periods. Second, our original data analysis plan did not include an explicit plan to stratify by vaping status, and relatedly, the size of our sample was based on power calculations that assumed a whole sample rather than a stratified analysis. The post hoc nature of those stratified analyses should be considered when interpreting the findings of this study. In addition, the Truth Initiative content used for the stimulus had previously aired, and prior exposure may have limited its treatment potential. Finally, although the observed effects of the intervention occurred among current vapers, the study was not powered by subgroups. Finally, we performed multiple comparisons in our analyses, which raises the possibility of false-positive findings. Future studies should use previously unaired content, where possible, and ensure a sufficient sample size among specific subgroups of interest, where feasible (ie, power at the subgroup level).

### Conclusions

Social media interventions are a promising approach to preventing vaping among young adults [13]. More research is needed on how to optimize the dosage of such interventions and the long-term effects on vaping use over time. Social media–based research platforms are a promising methodology to conduct experimental public health research among specific priority populations.

## Appendix

Multimedia Appendix 1 Comparison of recruited and retained samples.

Multimedia Appendix 2 CONSORT eHealth Checklist (V 1.6.1).

## Abbreviations

CONSORT: Consolidated Standards of Reporting Trials
FM: Facebook Messenger
FU: follow-up
IRB: Institutional Review Board
LTFU: lost to follow-up

## Data Availability

A data sharing agreement is required for the use of all data. Our research team does not share data with tobacco industry representatives or affiliated researchers. Investigators seeking access to the data used in the study should make a written request to the corresponding author and submit a detailed research plan including the purpose of the proposed research, required variables, duration of the analysis phase, institutional review board approval, Federalwide Assurance information, and documentation of investigator training in human participants.
