# Supplementary material for: Effects of a Social Media Intervention on Vaping Intentions: Randomized Dose-Response Experiment
Source: J Med Internet Res. 2024 Mar 12;26:e50741. doi: 10.2196/50741 (PMC10966440; doi:10.2196/50741)
Supplement: Multimedia Appendix 1 [file jmir_v26i1e50741_app1.pdf]

Supplemental Table. Comparison Between Recruited & Retained Samples

	Recruited (n=1,491)	Retained (n=730)
	n (%)	n (%)
**Age**		
18	199 (13.35)	92(12.60)
19	210 (14.08)	97(13.29)
20	200 (13.41)	99 (13.56)
21	212 (14.22)	105 (14.38)
22	223 (14.96)	107 (14.66)
23	227 (15.22)	109 (14.93)
24	220 (14.76)	121 (16.58)
**Race/ethnicity**		
White, non-Hispanic	710 (47.62)	351 (48.08)
Black, non-Hispanic	118 (7.91)	46 (6.30)
Hispanic	257 (17.24)	134 (18.36)
Other, non-Hispanic	406 (27.23)	199 (27.26)
**Sex**		
Female	1,027 (68.88)	494 (67.67)
Male	409 (27.43)	214 (29.32)
Another identity/Non-Binary/Transgender	55 (3.69)	22 (3.01)
**Perceived financial situation**		
Lives comfortably	490 (32.86)	248 (33.97)
Meets needs with a little left over	488 (32.73)	235 (32.19)
Meets basic expenses	416 (27.90)	201 (27.53)
Doesn’t meet basic expenses	97 (6.51)	46 (6.30)
**E-cigarette use at baseline**		
Never users	841 (56.71)	411 (56.30)
Former users	331 (22.32)	171 (23.42)
Current users	311 (20.97)	148 (20.27)
**Sexual orientation**		
Heterosexual	936 (70.32)	459 (69.97)
Bisexual	234 (17.58)	115 (17.53)
Homosexual	93 (6.99)	46 (7.01)
Asexual	28 (2.10)	14 (2.13)
Another sexual orientation	40 (3.01)	22 (3.35)
